# Investigation and estimation of the prevalence of drug addicts in Xichang, china

**DOI:** 10.1097/MD.0000000000013919

**Published:** 2019-01-04

**Authors:** Kejin Chen, Chaorong Bian, Benli Song, Ge Gao

**Affiliations:** a Changzhou Maternal and Child Health Care Hospital, Changzhou; b Changzhou Children's Hospital, Changzhou; c Xichang dermatology and venereal disease prevention and control station; d Department of Epidemiology and Biostatistics, School of Public Health, Medical College of Soochow University, 199 Renai Road, Industrial Park, China.

**Keywords:** capture-recapture analysis, estimation of prevalence, injecting drug-users (IDUs), reliability and validity

## Abstract

To use a new method to estimate the prevalence of drug addicts in Xichang for the first time and to suggest that Chinese Center for Disease Control and Prevention should strengthen surveillance and intervention for them.

The Capture-Mark-Recapture (CMR) method of 3 correlated sources under the simple random sampling and its formulas detailed in this paper were applied to a survey of issues relevant to drug addicts in Xichang, China.

To view half a year as reacquisition time, the prevalence of drug users in Xichang could be 5264 in 2014, accounting for 0.7% of the resident population of the same year.

This investigation method and its formulas studied in this paper have both high reliability and high validity, being worthy of promotion in terms of estimating the prevalence of drug addicts in other parts of the world.

## Abstract 2

Supplemental Digital Content is available in the text

## 1 Introduction

The drug problem is a serious social problem in the world we face today. Drug addicts have become a group characterized by high-risk behavior, as it involves the spread of many infectious diseases including Acquired Immune Deficiency Syndrome (AIDS), hepatitis, tuberculosis and sexually transmitted diseases. The size of high-risk behavior population is the basis of analysing epidemiological situations, formulating intervention measures and allocating resources for prevention and control.^[1]^ In recent years, the Chinese government stresses crackdowns on drug-related crimes, however, due to various factors, drug abuse and drug-related crime have not been reduced. What's more, types of abused drugs have become increasingly diversified, especially in small and medium-sized cities and rural areas.

According to the data provided by Sichuan center for disease control and prevention and Xichang dermatology and venereal disease prevention and control station, the number of injected drug users in Xichang is 2400 in 2000, 2610 in 2002 and 2800 in 2004.^[2,3]^ Meanwhile, behind each registered drug user, there are implicitly about 2 to 4 drug users,^[4]^ therefore, we believe that the prevalence of drug abusers will increase if unregistered drug users are included.

Owing to that Xichang is the state capital of Yi Autonomous Prefecture of Liangshan, the crowded lifestyle is mainly affected by the Yi people's customs. As a prominent feature of the Yi customs, the Yi people regard widespread drug use as a kind of ‘aristocratic deal’.^[5]^ In addition, the whole group of drug addicts receives very little social support because they have been on a lower social level for a long time. In the context of the widespread existence of social discrimination, their self-esteem is seriously frustrated. And even some drug addicts tend to seek self-affirmation and vent their emotions in the commercial sexual activity.^[6]^ So there is an urgent need for comprehensive interventions in this region to curb the rapid growth of drug addicts, particularly expanding the coverage of methadone maintenance treatment (MMT) programs and estimating the prevalence of drug addicts more accurately.^[7]^

Xichang MMT clinic is one of the 8 outpatient departments which belongs to the first batch of methadone pilot project approved by the Chinese government in 2003. A prospective cohort study followed up for 6 years, involving 280 patients who had been treated with methadone maintenance in Xichang, declared that the treatment retention rate was only 39.6%, resulting from low therapeutic dose, stealthily do drug and without family members escort during treatment.^[8]^

Many researchers have carried out the relevant work to explore the method of estimate the size of high-risk behavior population, but they never have standard estimation methods and guidelines in the technical documents owing to the sensitivity and specificity of high-risk behavior population. Under these circumstances, they cannot provide reliable and effective data about the prevalence. Meanwhile, after perusing domestic and foreign articles, we find that the studies on drug addicts almost focus on the infection rate, risk factors and behavioral interventions. In recent 10 years, there has not been a study on the prevalence of drug addicts in Xichang.

## 2 Methods

### 2.1 Incomplete table

Data from 3 sources can be organized into an incomplete table containing the grid with a missing datum (Table 1). *x*_*ijk*_ in the table is the number of corresponding objects observed. Subscripts *i*, *j*, and *k* respectively represent that whether they are from *i*, *j*, and *k* sources or not. If it belongs to that source, the value of corresponding subscript will be 1; if not, the value will be 2. *x*_222_ represents that they do not appear in all 3 sources. The probability that the observed object appears in the *i*, *j* and *k* lattice is denoted by *p*_*ijk*_.

**Table 1:**
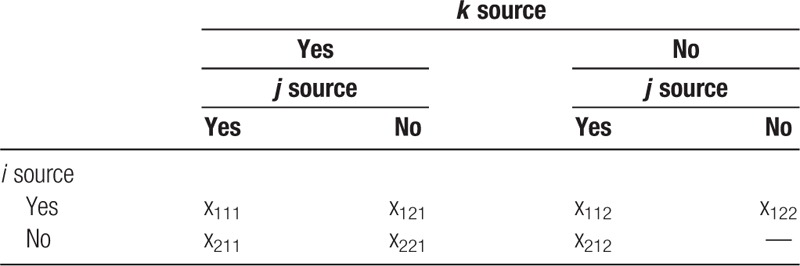
2^3^ incomplete table from 3 sources of CMR method. CMR = Capture-Mark-Recapture.

### 2.2 Statistical model

There are only 7 lattices filling with observation data, so more than 7 parameters cannot be fitted with this model. If we check the logarithmic linear model for desired value {*m*_*ijk*_} which is the theoretical estimate of *x*_222_, we cannot measure 3-factor effect from 3 sources.

According to the characteristics of high-risk groups, we usually use the logarithmic linear model in which there is a link between each pair of sources: 
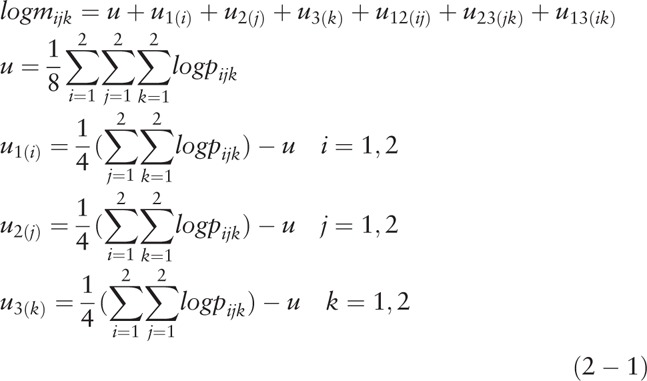


*u* stands for the mean of the probabilistic logarithm values in each grid. *u*_1(*i*)_, *u*_2(*j*)_ and *u*_3(*k*)_ represent the deviation of *u*.

*u*_12(*ij*)_ indicate the deviation of 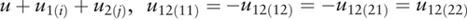.

*u*_23(j*k)*_ indicate the deviation of 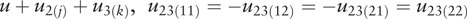.

*u*_13(*ik*)_ indicate the deviation of 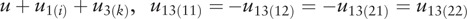.

If 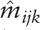 is calculated, we can evaluate whether the model fitting by observed value is appropriate as follows: 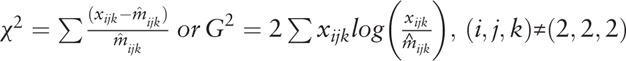


### 2.3 Estimation of total quantity

For the log-linear model previously described, maximum likelihood estimate of expected value can be obtained by that the sum of *u* in the highest order is equal to their corresponding observed values. After knowing the condition of estimating {*m*_*ijk*_}, it is easy to get {*p*_*ijk*_}. We assume that the number of individuals that do not appear in the 3 sources is *m*_222_. The Bartlett criterion with no 3-factor effect is applied to the log-linear model above, so we can calculate as follows:
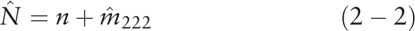

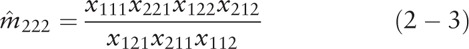


### 2.4 Estimation of variance


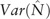
, the asymptotic variance of 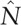 can be calculated using vertical projection method: 
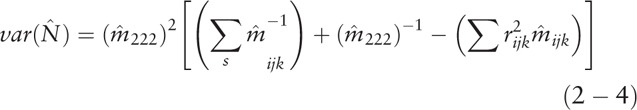


*S* represents the sum of the corresponding number of the other 7 grids except for the 1 with missing data in Table 1.
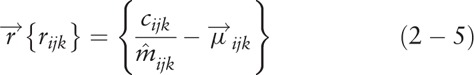

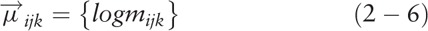


To choose 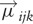 to make 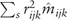 reach the minimum, when the sum of *i*, *j*, *k* is odd, 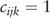; when the sum is even, 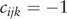.

95% confidence interval of total quantity can be calculated as follows: 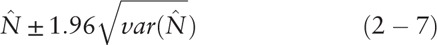


### 2.5 Study population

Objects in the study must meet the following conditions:

1. Residents with the household registration of Xichang or residents unmoved more than 6 months in the geographical scope of Xichang.
2. Active drug users: the drug abusers who have been deprived of drug addiction for less than 3 years as of July 31, 2014, consisting of the ones still in social activities, the ones who have been forced into drug rehabilitation centers or voluntarily detoxified drugs, the ones who have been re-educated through labor and imprisoned no more than 3 years.
3. Mainly heroin users including the ones simply using heroin, the ones using mixed drugs such as the combination of heroin and diazepam, heroin addicts receiving MMT.

The cases we get are from 3 kinds of sources in Xichang, namely 4 drug rehabilitation centers, 1 MMT clinic located in the urban area, 5 methadone treatment extension point in rural areas (communities) respectively. The participants provided their written informed consent to participate in this study anonymously. The data were also collected and analyzed anonymously.

### 2.6 Data management

All the participants had signed the informed consents. The questionnaires were completed by respondents independently and all of the questionnaires were checked carefully. The questionnaire consists of 5 parts, 20 items, presented in Supporting Information Questionnaire S1. Some item can be evaluated as “yes” or “no”. We consider respondents who were to one or other of the 3 sources in the last 6 months through questionnaires.

In this study, 1007 questionnaires were distributed, of which 644 in the drug rehabilitation center, 178 in the community and 185 in the methadone clinics. Finally, 1007 questionnaires were collected. Both the rate of collecting questionnaires and the qualified rate are 100%. The collected data was checked twice and used to set up the database with EpiData 3.1. SAS9.3 was used to analyze the data.

## 3 Results

### 3.1 General analysis

Among 1007 respondents in the survey, male accounted for 84.0% (846/1007), the average age was 28.2 ± 7.0. The Han nationality and the Yi accounted for 43.7% (440/1007) and 56.3% (567/1007) respectively.

In the past 6 months before the inclusion, only 242 cases (24.03%) of drug addicts received free condoms, 835 cases (82.92%) received education and behavior intervention about Human Immunodeficiency Virus (HIV)/AIDS, 537 cases (53.33%) received HIV antibody testing. It is worth noting that 312 cases (85.95%) did not receive drug detoxification among 363 cases of drug addicts from the methadone clinics and the community.

With regard to the use of drugs (Table 2), we found that, among 1007 cases in the past 6 months, 729 cases (72.39%) used heroin at least once a week and 594 cases (58.98%) of them used once a day and above. Moreover, 235 cases (23.33%) used methadone at least once a day and 70 cases (6.95%) used methamphetamine at least once a week. The proportion of using ecstasy, ketamine, marijuana, pethidine (dolantin), opium, morphine were all less than 2%. No one has ever used nitrite.

**Table 2:**
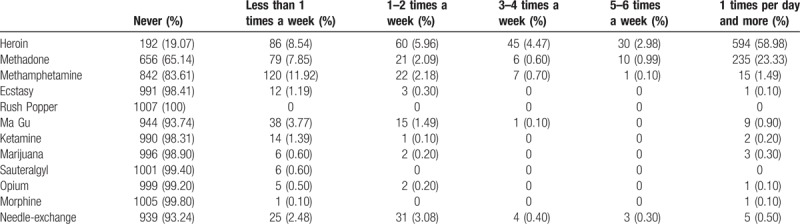
Their situation of drug taking in the past 6 months.

### 3.2 Prevalence of drug addicts

We organized the results of the survey to consider the respondents who have been to the drug rehabilitation centers, the communities and the methadone clinic in the past 6 months or not as the first, second and third samples respectively. And then the results were summarized into a 2^3^ incomplete table with a missing grid (Table 3).

**Table 3:**
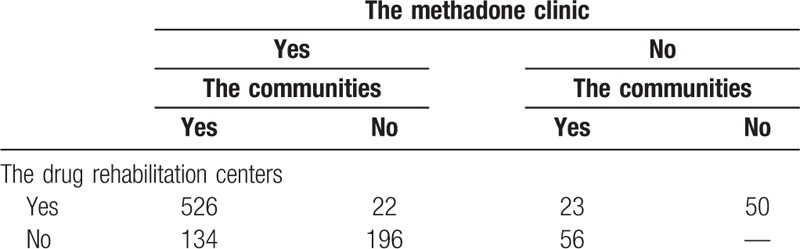
2^3^ incomplete table from 3 sources of drug addicts in Xichang.

The Capture-Mark-Recapture (CMR) model of 3 correlated sources under simple random sampling was served to estimate the prevalence of drug addicts in Xichang. According to the relevant data in Table 2 and the formulas (2–2), (2–3), we can reckon up the prevalence as follows: 
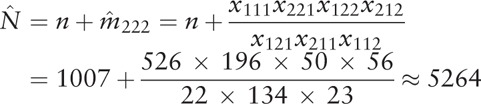


On the basis of orthogonal projection method in different inner product space in the formula (2–4), and after iterative calculation of SAS programming, we can figure out the asymptotic variance of 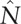: 
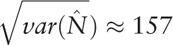


So 95% confidence interval (95% *CI*) of the total quantity of drug addicts in Xichang 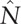 is: 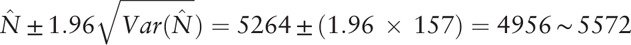


In the light of Bureaus of Statistics in Xichang, the resident population of Xichang in 2014 reached 0.751 million.^[9]^ That means the size of drug addicts in Xichang make up 0.66∼0.74%of residents there.

### 3.3 Evaluation of reliability and validity

In order to evaluate the reliability and validity of the CMR method of 3 correlated sources under the simple random sampling and its formulas in our study, the statistic values worked out by survey data about drug addicts in Xichang in 2014 were regarded as simulated population parameter (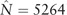). By means of Monte Carlo simulation and SAS programming, we repeated simulated sampling of 100 samples for the CMR method of 3 correlated sources and its formulas. The sample sizes for 3 categories of each sample were 644, 178, and 185 respectively. In the light of the formulas in front and SAS programming, we can get my hands on the data about total quantity estimates of 100 samples and their homologous 95% confidence intervals.

**Figure 1:**
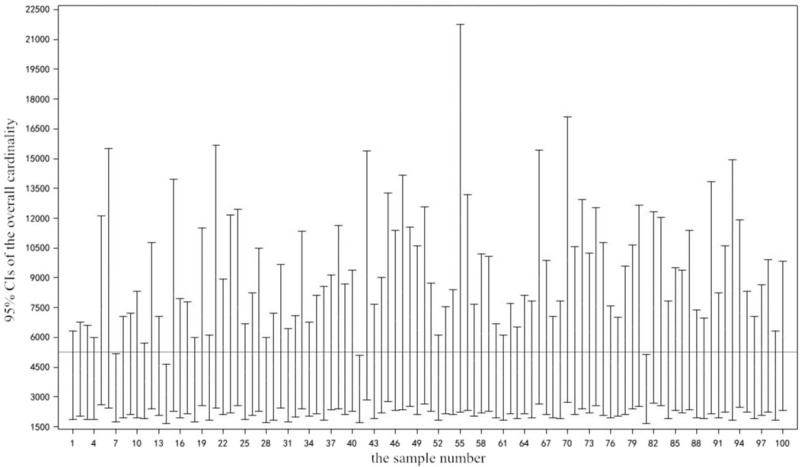
The computer simulation results of the CMR method of 3 correlated sources. CMR = Capture-Mark-Recapture.

The sample number and the 95% confidence interval of each sample were taken as the abscissa and the ordinate respectively, as well as that simulated population parameter (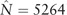) was prescribed as the baseline, constitute the figure as below. From Figure 1, we know that only 4 times do not contain the simulated total quantity (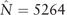) among the 95% confidence intervals of the total quantity obtained from 100 simulations. The results show clearly that this investigation method and its formulas have both high reliability and high validity, being worthy of promotion in medical research.

## 4 Discussion

The drug users in the communities have better sample representativeness, but due to their covert behavior, it is not easy to find enough samples and conduct investigation. And for the drug users in the drug rehabilitation centers, their willingness to cooperate can make samples easy to obtain but lack of representativeness. The proportion of being arrested by public security departments or being forced to detoxification among the friends of drug addicts in the drug rehabilitation centers could be higher than that in the communities. Estimates of prevalence of drug addicts who are obtained from the drug rehabilitation centers alone may cause an underestimation of the size of drug addicts.^[10]^ Therefore, our team selected 3 types of places (drug rehabilitation centers, communities, methadone clinic) as 3 sources in our study to obtain enough samples and ensure representativeness of samples.

Like many other statistical models in the field of scientific research, CMR model is based on some assumptions. In order to facilitate the study, scholars often make a series of assumptions. Due to the conditional constraints, they can only assume that the observed situation is consistent with what is not observed. Certainly, the hypothesis cannot be completely out of line with the actual, and should be the same as the actual situation as much as possible, otherwise, the theoretical model based on the assumption loses its meaning. If there is a difference between the reality and the hypothesis, 3 things have to be done: discovering all the divergences between the actual and the hypothesis, assessing the severity of the divergences, finding a solution to the problem.^[11]^ However, if the difference exceeds the critical criteria applicable to the model, we need to consider another model. For example, if the assumption of general closure is not invalid, we should use the open-ended Cormack-Jolly-Seber model; if the hypothesis of individual homogeneity is not established, the population should be stratified first, then drug users who own the same or similar probability of capture should be grouped into the same layer, and we need to use CMR model independently in each layer at last; if the supposition of independence is not supported, it is necessary to use a log-linear CMR model of multi-samples.^[12]^

According to the plan issued by Liangshan Prefecture, “Work Programme of Police Officers of Politics and Law Stationed in the Villages”,^[13]^ in 2013, the state selected 200 plainclothes policemen from the political-legal department, and conducted investigations in 180 key villages with drug trafficking and drug abuse. In order to effectively control the drug-related personnel, these plainclothes police officers united some villagers and gradually knew and mastered the drug-related situation in the villages. They regularly reported information on drug users, drug traffickers and potential drug users to the drug control departments. According to the data provided by the drug control department in Xichang, the number of drug users registered in Xichang in 2014 was 3036, and the number of drug users actually mastered was 5313. The estimate “N = 5264” is close to the real prevalence. By comparison, we can easily find that, for the estimate of prevalence of drug addicts in Xichang, not only the theory is scientific, but also the practice is feasible.

The Delphi method is mainly used for the problems such as insufficient historical data, incomplete objective data, many unmeasured influencing factors, strong policies, specific and single dimension, or for the estimation process that is difficult to predict by other forecasting methods.^[14]^ Before using the multiplier method to estimate the population size, the actual conditions on the site should be evaluated in conjunction with the available information. In the case of that the multiplier cannot be obtained, a special epidemiological supplementary survey is required. The application of multiplier method requires attention to whether the target population is clearly defined, whether the survey sample is representative, that is, whether to match the time, region, and sample, whether the base number is accurate, and whether the proportion of contact is true and reliable.^[15]^

In fact, the assumptions of the CMR method and the multiplier method are equivalent, except that the CMR method utilizes more information and regularly relies on existing data from multiple sources. The Lincoln-Petersen model is the simplest 2-source model of the CMR method. Using the CMR method to estimate the prevalence rate based on the single source of compulsory detoxification centers, it is necessary to define the relevant indicators and concepts of capture and capture interval. Improving the quality of data from single source and using parallel data from multiple sources to promote the methodology are the promising research fields in the next few years.^[16]^

In the survey, if the respondents have great mobility, the probability of capturing the target population who were marked at the first capture would be reduced, resulting in the overestimation of the result. The impact of the public security crackdown is a double-edged sword. On the one hand, it could lead to a decrease in the target population that could be captured on the day of the investigation, resulting in underestimation of the result; on the other, it could also lead to a decrease in the probability of recapture, overestimating the result.^[17]^

Before estimating the total quantity, we must first clarify that why we carry out the survey and how to use the outcome. The size of high-risk population can provide local government with very important information, but this does not mean that estimating the population size could be launched in any region regardless of any conditions. What we need to do is combine the actual situation in the local place and meet the current needs as the prerequisite.

For the injection drug users in Xichang, the primary route to be infected with HIV is through sharing syringes.^[3]^ A prospective cohort study about local IDU was carried out to reveal that developing a program of exchanging syringes could reduce the infectivity of HIV infected persons.^[2]^ Sex workers of IDU have an important impact on transmission and epidemics of HIV/AIDS,^[18]^ but among IDU in Xichang, the possibility of sexually transmitted HIV is small.^[19,20]^ Compared to HIV, Syphilis infection is more serious and taking on ascend trend,^[21]^ so there is an urgent need for behavioral interventions and venereal treatment of intravenous drug users, especially for women and ethnic minorities, to control the spread of syphilis in this population.

There is significant efficacy of the first group of MMT clinic in China in reducing drug using and sexual risk behaviors, improving social function and family relationship.^[22]^ However, MMT retention is low in China, intervention service and education should be provided to MMT patients to increase retention and to community to reduce discrimination, respectively.

The results of our research have attracted the attention of the Xichang Municipal People's Government. In 2015, the government officially promulgated documents, that is, “Incentives to Report Drug Crimes in Xichang”. This move has been strongly supported by the broad masses of the people. They have taken the initiative to report drug-related crimes to public security organizations and provide clues for solving crimes.^[23]^ In Liangshan Prefecture, civilian drug control work is mainly carried out in the form of anti-drug association. For example, the anti-drug associations have covered 271 villages in Zhaojue County, involving 152,000 people of 96,000 households, accounting for 47.46% of the total population of the county (152,000/320,300, inb2016). At present, among the 170,000 drug addicts registered in Sichuan Province, 75% are under 35 years old. Meanwhile, the number of drug abusers under the age of 17 has increased rapidly, and has become a major trend in the past 5 years.^[24]^ The lack of correct values education from childhood is the main reason for this phenomenon, so it is especially important to increase the publicity and education of drug control at school age.

## 5 Conclusions

In this paper, we elaborate on the CMR method of 3 correlated sources under simple random sampling and its statistical model. We apply orthogonal projection method to estimate the asymptotic variance of total quantity, making the estimation more accurate. Our team designed a particular questionnaire, and then 1007 existing drug addicts were taken out randomly from drug rehabilitation center, community, methadone outpatient in Xichang as the respondents. As a result, we have gained a lot of information about HIV prevention in Xichang, and what's more, scientifically worked out the prevalence of drug users there.

### 5.1 Ethics approval and consent to participate

Not applicable. On the basis of patients’ informed consent, we only conducted questionnaires, thus no ethical approval are required.

### 5.2 Consent to publish

This manuscript is approved by all authors for publication.

### 5.3 Availability of data and materials

All the data and materials can be available in Supporting Information enclosed in Submission System, namely Questionnaire S1, 1007 cases of results, SAS simulation program and 100 simulation results.

## Acknowledgment

We would like to thank Qiaoqiao Du, Ying Fu, and professor Dabing Lv, for their help and encouragement. We also thank the referees and editors for their suggestions and help.

## Author contributions

**Conceptualization:** Kejin Chen, Ge Gao.

**Data curation:** Kejin Chen, Benli Song.

**Formal analysis:** Kejin Chen, Chaorong Bian.

**Funding acquisition:** Ge Gao.

**Investigation:** Kejin Chen, Benli Song.

**Methodology:** Kejin Chen, Ge Gao.

**Project administration:** Kejin Chen, Benli Song.

**Resources:** Kejin Chen, Chaorong Bian.

**Software:** Kejin Chen, Chaorong Bian.

**Supervision:** Ge Gao.

**Validation:** Ge Gao.

**Visualization:** Kejin Chen.

**Writing – original draft:** Kejin Chen.

**Writing – review & editing:** Kejin Chen, Ge Gao.

## Supplementary Material

Supplemental Digital Content
