# Supplementary material for: Investigation and estimation of the prevalence of drug addicts in Xichang, china
Source: Medicine (Baltimore). 2019 Jan 4;98(1):e13919. doi: 10.1097/MD.0000000000013919 (PMC6486045; doi:10.1097/MD.0000000000013919)
Supplement: Supplemental Digital Content [file medi-98-e13919-s002.doc]

**Rapid assessment of drug users**

Note:

- Please mark “×” when you select.
- Not included this time or have ever participated.

ID number: ■■■■■■□□□□□□□□□□□□

Nationality: 1、Han □ 2、Yi □

A．Source of respondents:

1、the drug rehabilitation center □

【If you choose the drug rehabilitation center, please directly jump to the C item】

2、the methadone clinic □ 3、the community □

B．Over the past 6 months, whether or not you abandoned drug habits in the drug rehabilitation center?

1、Yes □ 2、No □

C．Over the past 6 months, have you received any of the following preventative interventions about AIDS?

【If you are from the drug rehabilitation center, please according to your condition 6 months before being sent to the drug rehabilitation center】

| C1. | publicity and education and behavioral interventions about AIDS | Yes □ | No □ |
| --- | --- | --- | --- |
| C2. | free condoms | Yes □ | No □ |
| C3. | HIV antibody testing | Yes □ | No □ |

D．Choose the appropriate frequency of the following drugs you used and what you do over the past 6 months.

【If you are from the drug rehabilitation center, please according to your condition 6 months before being sent to the drug rehabilitation center】

|  |  | Never | Less than 1 times a week | 1-2 times a week | 3-4 times a week | 5-6 times a week | 1 times per day and more |
| --- | --- | --- | --- | --- | --- | --- | --- |
| D1. | Heroin | 1、□ | 2、□ | 3、□ | 4、□ | 5、□ | 6、□ |
| D2. | Methadone | 1、□ | 2、□ | 3、□ | 4、□ | 5、□ | 6、□ |
| D3. | Methamphetamine | 1、□ | 2、□ | 3、□ | 4、□ | 5、□ | 6、□ |
| D4. | Ecstasy | 1、□ | 2、□ | 3、□ | 4、□ | 5、□ | 6、□ |
| D5. | Rush Popper | 1、□ | 2、□ | 3、□ | 4、□ | 5、□ | 6、□ |
| D6. | Ma Gu | 1、□ | 2、□ | 3、□ | 4、□ | 5、□ | 6、□ |
| D7. | Ketamine | 1、□ | 2、□ | 3、□ | 4、□ | 5、□ | 6、□ |
| D8. | Marijuana | 1、□ | 2、□ | 3、□ | 4、□ | 5、□ | 6、□ |
| D9. | Sauteralgyl | 1、□ | 2、□ | 3、□ | 4、□ | 5、□ | 6、□ |
| D10. | Opium | 1、□ | 2、□ | 3、□ | 4、□ | 5、□ | 6、□ |
| D11. | Morphine | 1、□ | 2、□ | 3、□ | 4、□ | 5、□ | 6、□ |
| D12. | Needle-exchange | 1、□ | 2、□ | 3、□ | 4、□ | 5、□ | 6、□ |

E．Over the past 6 months, have you ever been to any of the following places?

【If you are from the drug rehabilitation center, please according to your condition 6 months before being sent to the drug rehabilitation center】

| E1. | Have you ever been to the drug rehabilitation center? | Yes □ | No □ |
| --- | --- | --- | --- |
| E2. | Have you ever been to the methadone clinic? | Yes □ | No □ |
| E3. | Have you ever been to the community? | Yes □ | No □ |

Questionnaire reviewers: ____________ Date: ____________

data fy1;

x111=526;x121=22;x211=134;x221=196;x112=23;x122=50;x212=56;

sample_size=sum(x111,x121,x211,x221,x112,x122,x212);

p111=x111/sample_size;

p121=x121/sample_size;

p211=x211/sample_size;

p221=x221/sample_size;

p112=x112/sample_size;

p122=x122/sample_size;

p212=x212/sample_size;

total_number=5264;

_nsize_=sample_size;

fuying=1;

run;

data fy2;

set fy1;

seed=1;

do i=1 to total_number;

call rantbl(seed,p111,p121,p211,p221,p112,p122,p212,investigation);

output;

end;

keep investigation fuying;

run;

proc surveyselect data=fy2 noprint

method=srs out=fy3 seed=1

sampsize=fy1 reps=100;

strata fuying;

run;

proc freq data=fy3 noprint;

table replicate*investigation/out=fy4(drop=percent);

run;

proc transpose data=fy4 out=fy5(drop=_name_ _label_ rename=(_1=x111

_2=x121 _3=x211 _4=x221 _5=x112 _6=x122 _7=x212));

by replicate;

id investigation;

run;

data fy6;

if _n_=1 then set fy1 (keep=total_number);

set fy5;

n_hat=(x111+x121+x211+x221+x112+x122+x212)+(x111*x221*x122*x212)/(x121*x211*x112);

n_var=(((x111*x221*x122*x212)/(x121*x211*x112))**2)*(1/x111+1/x121+1/x211+1/x221+1/x112+1/x122+1/x212+(x121*x211*x112)/(x111*x221*x122*x212));

upper_limit=n_hat+1.96*sqrt(n_var);

lower_limit=n_hat-1.96*sqrt(n_var);

if lower_limit<total_number<upper_limit then status='p>0.05';

else status='p<0.05';

run;

proc print data=fy6;

var replicate n_hat n_var lower_limit upper_limit status;

run;

proc freq data=fy6;

table status;

run;

data fy7;

set fy6;n_hat=round(n_hat,1);upper_limit=round(upper_limit,1);lower_limit=round(lower_limit,1);

run;

data test1;

set fy7;

samp=replicate;

ref=5264;

run;

ods graphics / reset noborder width=1000px height=618px

imagename='计算机模拟结果' imagefmt=png noscale;

proc sgplot data= test1;

title1 "计算机模拟结果" ;

xaxis values=(1 to 100 by 3) label="样本序号" offsetmin=0.02 offsetmax=0.02;

yaxis values=(20963 to 439680 by 50000) label="总体基数估计的95%CI";

refline ref /axis=y;

scatter x=replicate y=n_hat /yerrorlower=lower_limit yerrorupper=upper_limit markerattrs=(size=0) ;

run;
